# Anti-Reflective Coatings Produced via Atomic Layer Deposition for Hybrid Polymer 3D Micro-Optics

**DOI:** 10.3390/nano13162281

**Published:** 2023-08-08

**Authors:** Darija Astrauskytė, Karolis Galvanauskas, Darius Gailevičius, Mantas Drazdys, Mangirdas Malinauskas, Lina Grineviciute

**Affiliations:** 1 Center for Physical Sciences and Technology, Savanorių av. 231, LT-02300 Vilnius, Lithuania; 2 Laser Research Center, Physics Faculty, Vilnius University, Saulėtekio av. 10, LT-10223 Vilnius, Lithuania

**Keywords:** atomic layer deposition, anti-reflective coating, micro-optics, SZ2080™, multi-photon lithography, direct laser writing

## Abstract

The increasing demand for optics quality requires the lowest optical power loss, which can occur from unwanted reflections. Laser direct writing (LDW) allows for the fabrication of complex structures, which is particularly advantageous in micro-optic applications. This research demonstrates the possibility of forming an anti-reflective coating on hybrid polymer micro-lenses fabricated by employing LDW without changing their geometry. Such coating deposited via atomic layer deposition (ALD) decreased the reflection from 3.3% to 0.1% at a wavelength of 633 nm for one surface of hybrid organic–inorganic SZ2080™ material. This research validates the compatibility of ALD with LDW 3D multiphoton lithography synergistically, expanding its applications on optical grade sub-100 μm scale micro-optics.

## 1. Introduction

**Figure 1:**
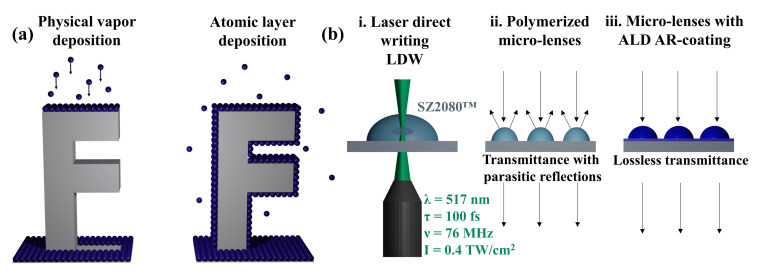
(**a**) The schematic representation of differences between physical vapor deposition and atomic layer deposition on the complex substrate; (**b**) fabrication of highly transparent micro-optics combining laser direct writing and atomic layer deposition.

Anti-reflective (AR) coatings are widely used in various optical applications to reduce unwanted reflections and enhance light transmission efficiency. These coatings are particularly beneficial in optical devices, such as lenses, mirrors, and displays, where reflections can cause glare, reduce contrast, and distort the image quality [1]. AR coatings are typically designed to minimize the reflection at a specific wavelength [2], several wavelengths [3], or over a range of wavelengths [4,5], depending on the application. Physical vapor deposition (PVD) techniques, such as electron beam evaporation, ion beam sputtering, magnetron sputtering, etc., are the most widely used methods for fabricating AR coatings, offering numerous advantages, including high deposition rates, good film uniformity, and the deposition of high-quality optical coatings with high laser-induced damage thresholds [6,7,8]. PVD techniques are commonly applied to deposit high-quality coatings on flat substrates or structured surfaces. Nonetheless, the ability to achieve conformal coating is limited to flat or modulated planar surfaces. Highly curved substrates or stacked structures cannot be uniformly coated. For instance, the ion beam sputtering method has been successfully utilized to deposit single-layer [9] and multilayer [10] coatings on nanostructured planar surfaces (gratings). However, significant progress has been made in designing and fabricating optical components over the past few decades. Multi-photon polymerization enables the fabrication of complex shape micro-optics, such as multi-lens micro-objectives [11], micro-lens arrays [12], free-form micro-lenses [12] or stacked gratings [13], which often suffer from reduced efficiency due to reflection losses. For such applications, PVD technologies are becoming insufficient and have limitations [14], such as poor conformality to cover micro and macro free-form optics (Figure 1a). In recent years, atomic layer deposition (ALD) has emerged as a promising alternative to PVD for fabricating coatings on complex shape 3D substrates [5,15,16,17,18]. ALD is a modified chemical vapor deposition (CVD) technique that allows precise film thickness control at the atomic level by sequentially exposing the substrate to alternating gaseous precursors. The process involves self-limiting surface reactions, where each precursor molecule reacts with the available reactive site of the substrate surface, forming a single atomic layer [19]. This makes it possible to achieve uniform and conformal coatings on complex substrates (Figure 1a).

This study aims to demonstrate the possibility of depositing AR coatings on micro-lenses with sizes less than 100 μm (Figure 1b) using ALD. The feasibility of the method is tested using a hybrid organic–inorganic polymer SZ2080™, which is compatible with the heat treatment post-processing [20], offering high optical damage resilience [21].

## 2. Materials and Methods

The hybrid organic–inorganic polymer SZ2080™ was selected for fabricating the 3D microstructures due to its favorable characteristics, including high optical transmittance and the ability to adjust the refractive index [22,23]. However, it is important to determine the dispersions of the refractive index and optical losses of SZ2080™ to model the design of the AR coating, as SZ2080™ is not a standard substrate for optical coatings, and its optical characteristics vary depending on fabrication parameters [12]. Before the deposition of the AR coating, it was necessary to evaluate the optical properties of the hybrid polymer SZ2080™; therefore, the hybrid polymer was spin-coated on a fused silica (FS) substrate to form a layer of few microns. Optical characteristics of SZ2080™ were determined from the transmittance spectra using the OptiChar 15.12 [24] software and used in the design of the AR coating. In order to investigate the growth dynamics of alumina on SZ2080™, the hybrid polymer was drop-casted on a quartz crystal sensor. Finally, the influence of the ALD-deposited coatings on micro-lenses geometry and optical function was studied.

### 2.1. Preparation of Spin-Coated Samples

To confirm the feasibility of AR coating on LDW-fabricated microstructures from hybrid organic–inorganic polymer SZ2080™ using ALD, polymer film properties were investigated. Such films are usually produced using UV curing while employing an appropriate photoinitiator (PI) [22]. During the film production, it was important to avoid adding any photoinitiator which would introduce time-dependent additional variability [25]. Therefore, we choose to perform thermal curing that tends to produce consistent degrees of crosslinking [26]. For SZ2080™, this approach is based on the spontaneous thermal polymerization of the methacrylic groups [27] and the heat-catalyzed condensation reaction of the inorganic network [22].

First, SZ2080™ was spin-coated on FS substrates using a Chemat Technology spin coater (9 s 600 RPM, 30 s 2000 RPM) and heat-cured at 120 °C for 2 h. Control samples were immersed in the developer methyl-isobutyl-ketone for 10 min to indirectly confirm the saturation of the crosslinking reaction. After annealing, the film thickness, refractive index and optical losses of the SZ2080™ layer were evaluated from the transmittance spectra.

SZ2080™ was also drop-casted onto the Inficon 750-1058-G10 quartz crystal sensor and then dried at 50 °C for 12 h. The lower temperature was chosen to allow for slow solvent evaporation without forming entrapped bubbles. Before quartz crystal microbalance (QCM) measurements, a prepared sample was placed in the ALD reactor and exposed to 20 sccm N_2_ flow for 12 h to stabilize the oscillations of the quartz crystal sensor.

### 2.2. Fabrication of 3D Microstructures

**Figure 2:**
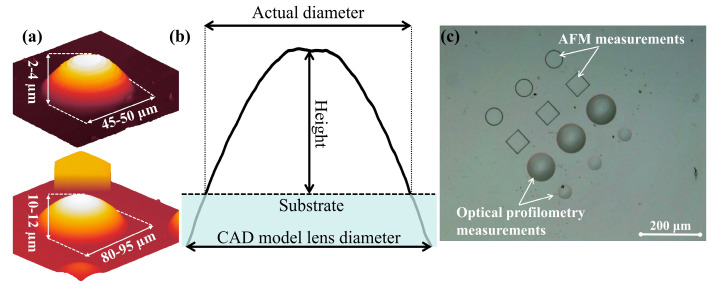
(**a**) Three-dimensional profiles of the fabricated ≈50 μm and ≈100 μm diameter micro-lenses; (**b**) comparison between modeled and fabricated micro-lens diameter; (**c**) optical micrograph of the 3D microstructures and micro-lenses fabricated using direct laser writing. These microstructures and micro-lenses were used for depositions of thin films.

In this work, flat surface microstructures and micro-lenses were fabricated via ultrafast 3D nanolithography conducted using direct laser writing. Microstructures were polymerized utilizing a laser oscillator with 517 nm wavelength, 100 fs pulse duration and 76 MHz repetition rate. Scanning was carried out with Femtika NanoFactory [28] system, utilizing an Aerotech IFOV technology known as continuous 3D writing [29]. Fabrication power was set to ≈3 mW, resulting in a ≈0.5 TW/cm^2^ intensity and a scanning speed of 1 mm/s. Three-dimensional models of the microstructures were developed using OpenSCAD 2021.01 [30]. At the same time, a fabrication machine code was generated using the 3DPoli 6.50 [28] software. Two models of lenses were used, with diameters of 50 μm and 100 μm, respectively, and focal lengths of 140–190 μm (Figure 2a). The final diameter and height of the polymerized lenses depend on the fabrication’s initial Z-stage position, which is set manually (Figure 2b). Flat structures—square and circular platforms (with dimensions of 50 μm × 50 μm × 5 μm)—were also fabricated and coated. Methyl-isobutyl-ketone (4-Methyl-2-pentanone) was used as a solvent to remove unpolymerized resist. Fabricated microstructures and micro-lenses are depicted in Figure 2c. Note that pure SZ2080™ hybrid material was without PI addition, as the femtosecond pulses can induce photopolymerization reactions directly via multiple excitation mechanisms, as revealed recently [31].

### 2.3. Deposition of Thin Films

Thin film deposition was performed using a Veeco Savannah S200 ALD system equipped with a capacitively coupled plasma generator. The depositions were carried out at 60 °C; 25.4 mm diameter FS substrates were used as substrates to deposit titania (TiO_2_) and alumina (Al_2_O_3_) thin films. Tetrakisdimethylaminotitanium (TDMAT) was used as a Ti-containing precursor and trimethylaluminum (TMA) was used as an Al source. TDMAT cylinder was heated to 75 °C to achieve an adequate vapor pressure, while TMA was kept at room temperature. Oxygen plasma was used as an oxidizer. Argon was used as a carrier gas at a flow of 40 sccm. For plasma generation, a 100 sccm flow of oxygen was used. All deposition parameters are summarized in Table 1.

### 2.4. Characterization Methods

The growth behavior of Al_2_O_3_ thin film on SZ2080™ was monitored using in situ quartz crystal microbalance. QCM enables the real-time observation of the stepwise thickness development of the growing thin film during the ALD process [32]. The growth per cycle can be calculated from the step height after one or other certain number of cycles. In this work, the growth per cycle was determined after each ALD cycle. The quartz crystal sensor was positioned in the center of the ALD reactor. For the characterization of the physical and optical properties of alumina and titania, single-layer coatings with a thickness of approximately 300 nm were deposited on FS substrates and LDW-fabricated 3D microstructures. Then, the single-wavelength AR coating consisting of 23 nm titania and 130.8 nm alumina was also deposited on both FS and microstructures, and the optical performance and physical characteristics were investigated. Transmittance and reflectance spectra were measured via a Photon RT Spectrophotometer in the spectral range from 185 nm to 2000 nm. Film thickness, refractive index and optical losses of the thin films were determined from the transmittance and reflectance spectra using the OptiChar 15.12 software. 

The stress in the thin films was calculated using the Stoney formula [33]. Interferometry was used to characterize the curvature radius of the FS substrates before and after the deposition of the thin films. The type of stress in the thin films was determined by observing the change in the substrate’s shape deformation after the depositions. Prior to depositions, the shape of the substrate was concave and after all depositions, the shape of the substrate was found to be more concave, indicating the presence of tensile stress in the thin films. 

Optical profilometry was used to measure the profiles of the micro-lenses before and after the deposition of different coatings (Figure 2c). The collected raw data were processed using the Gwyddion 2.63 software. Atomic force microscopy (AFM) was used to evaluate the surface morphology and roughness of the flat surface microstructures before and after the deposition of different coatings (Figure 2c). The scan area was 10 μm × 10 μm, with a line resolution of 512. The acquired raw data were analyzed using NanoScope Analysis 1.40 software.

The focal lengths of the micro-lenses were evaluated using an optical microscope via imaging a square aperture and a USAF 1951 resolution target. The imaging object was positioned on the microscope condenser diaphragm, essentially providing a virtual object at a distance significantly greater than the focal length of the micro-lens, and the image plane was adjusted until it was in focus. By placing the object at a distance much greater than the focal length, it could be assumed that the object is located at infinity and that the image being viewed is in the focal plane. The focal lengths of three ≈50 μm diameter and three ≈100 μm diameter micro-lenses were measured before and after the deposition of the thin films. The changes in the micro-lenses’ focal lengths were analyzed and the absolute errors were calculated. 

**Figure 3:**
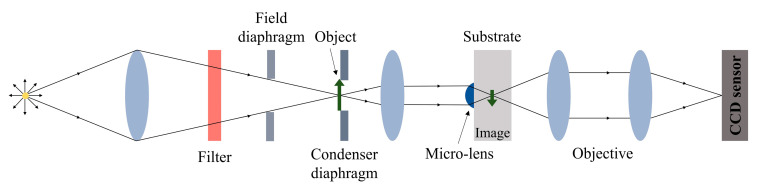
Optical system scheme for micro-lens image detection.

The relative transmittance of the micro-lenses was approximately determined by utilizing a square aperture as the object (Figure 3). An image of the aperture was captured using a filter that closely matched the AR coating spectrum. The CCD sensor operated in a grayscale mode and a fixed portion of the square image was integrated to obtain a numerical average of the gray color. No gamma correction or additional post-processing was applied, assuming that the obtained image brightness is linearly proportional to the amount of light transmitted. However, it is important to note that this method is only suitable for comparing elements of identical shape, as it provides relative transmittance values. The relative transmittance measurements were repeated on three different ≈ 95 μm diameter micro-lenses before and after the deposition of AR coating, and the absolute errors were calculated. For comparison, the transmittance of the three flat surface microstructures was measured and the errors were calculated.

## 3. Results and Discussion

### 3.1. Growth Dynamics of Aluminum Oxide on SZ2080™

**Figure 4:**
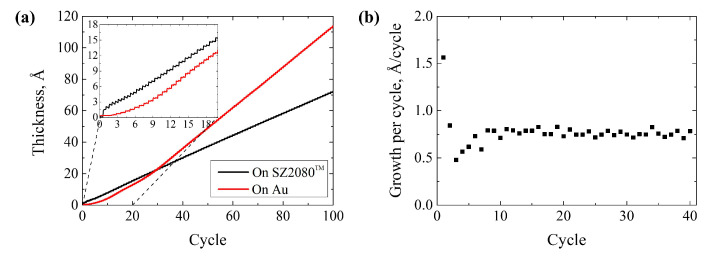
(**a**) Al_2_O_3_ film thickness as a function of the number of atomic layer deposition cycles measured via quartz crystal microbalance. The inset shows data collected in the first 20 cycles; (**b**) the growth per cycle of the Al_2_O_3_ as a function of the number of cycles determined from data collected through quartz crystal microbalance.

The growth dynamics of thin films strongly depend on the substrate, with the most significant differences occurring at the beginning of the film growth. The initial nucleation period plays a crucial role in producing continuous ultrathin films that could be utilized in the design of optical coatings [34]. Alumina growth dynamics on different polymers have been extensively studied [35,36,37], but to date, there has been no investigation conducted on hybrid organic–inorganic polymers such as SZ2080™. In this study, the growth dynamics of Al_2_O_3_ by ALD on the drop-casted hybrid polymer SZ2080™ were investigated using in situ QCM at 150 °C. Figure 4a shows aluminum oxide growth dynamics on SZ2080™ (black line). For demonstration purposes, growth dynamics on bare quartz crystal coated with Au are also shown (red line). During the first 50 cycles of aluminum oxide growth on quartz crystal, the growth rate is initially low due to a lack of nucleation sites, resulting in an island-like growth, as described by R. L. Puurunen and W. Vandervorst [38]. In the case of SZ2080™, in the first two cycles, the growth per cycle is initially high before rapidly decreasing and then increasing until reaching linear growth (Figure 4b). This behavior can be attributed to the porous surface of the hybrid polymer [39]. Linear growth started after eight cycles when continuous Al_2_O_3_ film was formed.

### 3.2. Spin-Coated and Thermally Polymerized SZ2080™ Film Optical Characteristics

**Figure 5:**
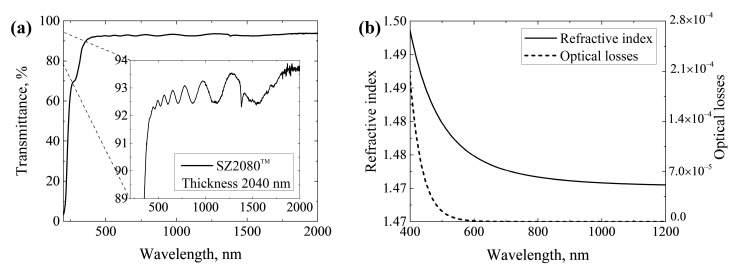
(**a**) Transmittance spectrum of spin-coated and thermally polymerized ≈2 μm thick SZ2080™ layer; (**b**) dispersions of refractive index and optical losses of spin-coated and thermally polymerized SZ2080™ layer.

Before the deposition of optical coatings, the optical properties of the spin-coated and thermally polymerized SZ2080™ film were characterized. The thickness of the SZ2080™ film was approximately 2 μm. Figure 5a shows the transmittance spectrum of the spin-coated SZ2080™ layer. The observed oscillations in this spectrum result from the interference phenomenon in which light waves reflected by a thin film’s upper and lower boundaries interfere with each other, either enhancing or reducing the reflected light. These oscillations depend on the thin film’s thickness and the material’s refractive index. Using the transmittance spectrum, the refractive index and optical losses of the SZ2080™ were simulated (Figure 5b). At a wavelength of 633 nm, the refractive index of the spin-coated SZ2080™ film is 1.48. Compared to the literature, a lower degree of crosslinking without the use of PI achieved via thermal polymerization can result in a lower refractive index [22,23,40].

### 3.3. Single-Layer Coatings

**Figure 6:**
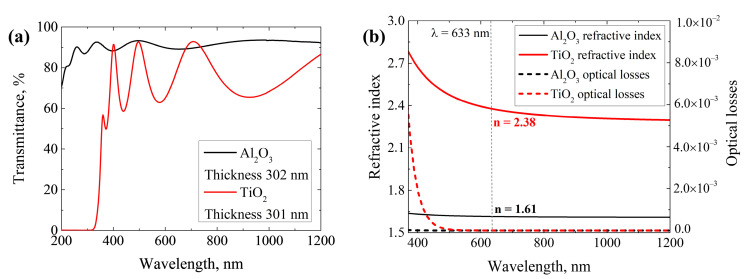
(**a**) Transmittance spectra of ≈300 nm thick Al_2_O_3_ and TiO_2_ thin films; (**b**) dispersions of refractive index and optical losses of Al_2_O_3_ and TiO_2_ thin films.

Alumina and titania were deposited on FS substrates to characterize the thin film’s optical properties, which will be used in the design of AR coating. Figure 6a shows the transmittance spectra of Al_2_O_3_ and TiO_2_ thin films with thicknesses of around 300 nm, while Figure 6b shows the dispersion curves of the refractive indices and optical losses. Refractive indices of TiO_2_ and Al_2_O_3_ at a wavelength of 633 nm are 2.38 and 1.61, respectively. Obtained optical properties of titania and alumina thin films are comparable with the previously reported study where the same precursors were used [41].

**Figure 7:**
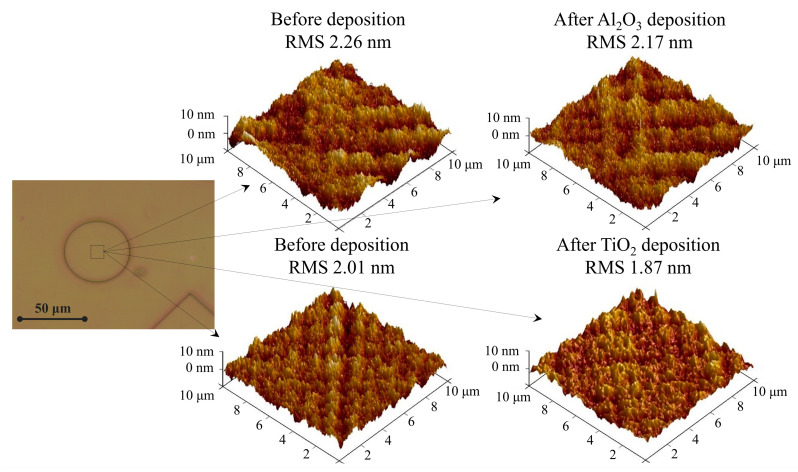
Three-dimensional atomic force microscope images of microstructures’ morphology before and after the deposition of Al_2_O_3_ and TiO_2_ films (scan area 10 μm × 10 μm).

After the characterization of the optical properties, physical characteristics of the thin films were analyzed. The surface roughness of LDW-fabricated microstructures was evaluated via AFM. Figure 7 depicts 3D AFM images of microstructures before and after the deposition of aluminum and titanium oxides. After the deposition of Al_2_O_3_ and TiO_2_ thin films, the surface roughness of the microstructures slightly decreased (see the explanation provided in Section 3.4).

**Figure 8:**
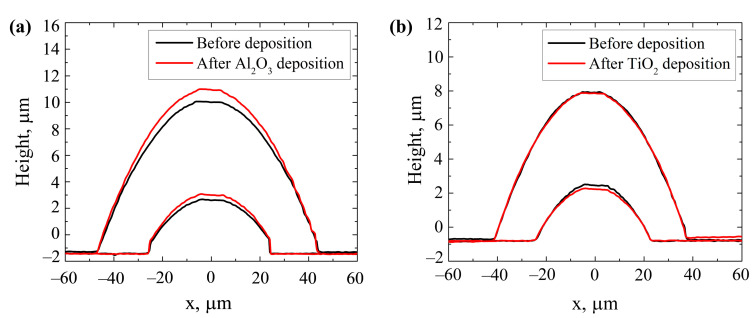
Profiles of the ≈50 μm and ≈80 μm diameter micro-lenses before and after the deposition of ≈300 nm thick (**a**) Al_2_O_3_; (**b**) TiO_2_ film.

Optical profilometry was used to determine the profiles of the micro-lenses. Figure 8 displays the profiles of the ≈50 μm and ≈80 μm diameter micro-lenses before and after the deposition of different single-layer coatings ((a) Al_2_O_3_, (b) TiO_2_). The focal lengths of three ≈50 μm diameter and three ≈ 80 μm diameter micro-lenses were measured before (*f*_0_) and after the deposition (*f*) of the thin films, and the averaged values are presented in Table 2, which also includes the averaged changes in the micro-lenses’ focal length (Δ*f*) after the depositions and the stress values of the thin films. As can be seen, changes in the micro-lens focal lengths correlate with the changes in the micro-lens height. In the case of aluminum oxide, focal length decreases by 7.5–8%, while the height of the micro-lenses increases. A different effect is observed after the deposition of TiO_2_—focal length increases by 2–7% and the height of the lenses decreases. Al_2_O_3_ and TiO_2_ thin films were determined to be under tensile stress with measured values of 135 MPa and 86 MPa, respectively. These findings are in agreement with previous studies, where ALD coatings typically exhibit tensile stress [42,43]. However, the relationship between the stress of the alumina coating and the changes in the micro-lens geometry is not clear. It can be assumed that the growth of the thin film influenced these changes. TMA molecules are initially smaller compared to TDMAT; consequently, these molecules can penetrate into porous polymer media, inducing an increase in the micro-lens height. Conversely, TDMAT molecules are significantly larger and cannot diffuse into the polymer. The observed decrease in the micro-lens height after the deposition of titania coating is caused by the tensile stress of the TiO_2_ thin film.

### 3.4. Anti-Reflective Coating 

**Figure 9:**
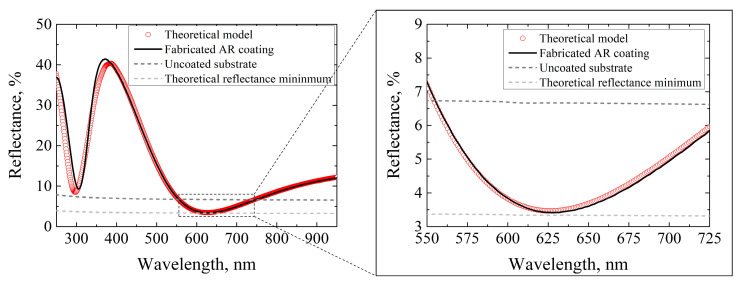
Simulated and experimental reflectance spectra of anti-reflective coating for λ = 633 nm.

As the optical properties of the substrate and individual layers were established, the design of the anti-reflective coating was simulated using TFCalc [44] (Figure 9, red curve). The AR coating was fabricated according to the theoretical model and consisted of 23 nm titanium oxide and 130.8 nm aluminum oxide layers. After the deposition, the reflectance of the experimental coating was measured (black line) in the spectral region of 200–950 nm. Discrepancies between the theoretical design and experimental curve in the UV range can be attributed to the absorption of TiO_2_. Compared to the theoretical transmittance minimum, AR coating reduced the reflectance of one SZ2080™ surface from 3.3% to 0.1% at a wavelength of 633 nm.

**Figure 10:**
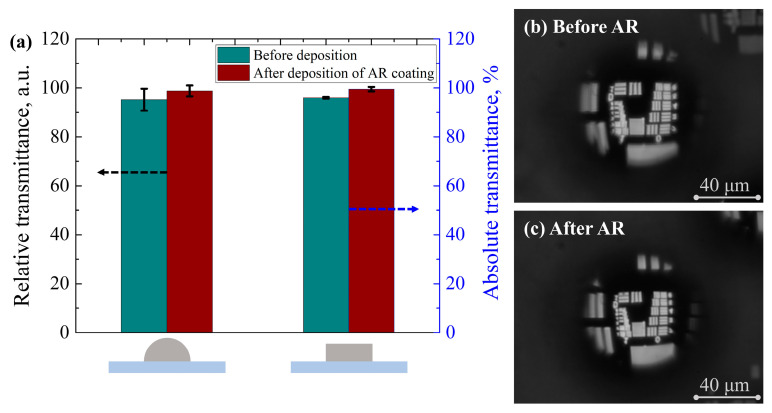
(**a**) Relative transmittance of the ≈95 μm diameter micro-lens and absolute transmittance of the flat surface microstructure before and after the deposition of anti-reflective coating at a wavelength of 633 nm. The error bars present calculated absolute error values; USAF image (**b**) before and (**c**) after anti-reflective coating deposition.

The same deposition process of AR coating was applied to the micro-lenses, but due to their small size, direct measurement of absolute transmittance was problematic. To overcome this, the relative transmittance of the ≈95 µm diameter micro-lenses was evaluated at a wavelength of 633 nm. The measurements were performed on three separate micro-lenses before and after the deposition of AR coating, and the averaged values are shown in Figure 10a. As can be seen, the relative transmittance of the micro-lenses increased by 3.7% after the deposition of the AR coating. In addition, the absolute transmittance of the flat surface microstructures was measured, and similar results were obtained with a 3.6% increase in transmittance. Also, an imaging quality comparison utilizing the USAF 1951 target was carried out on the micro-lenses, and the images before and after the deposition of the AR coating are shown in Figure 10b and Figure 10c, respectively. No changes in image quality were observed after the deposition of the AR coating.

**Figure 11:**
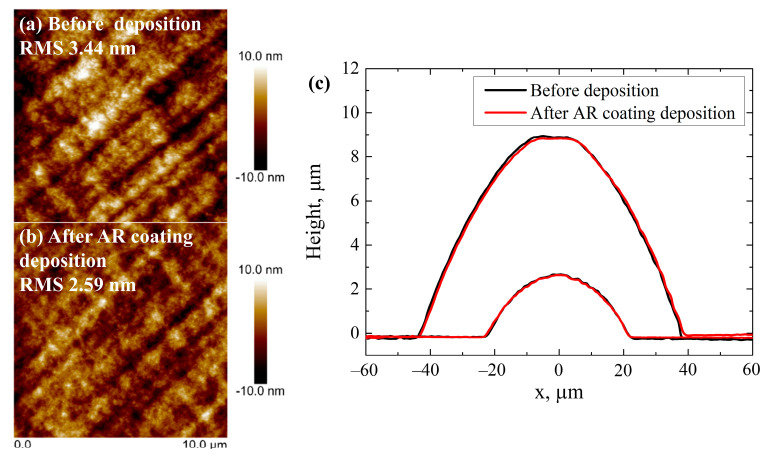
Surface morphologies of SZ2080™ microstructures measured with atomic force microscope (**a**) before deposition and (**b**) after deposition of the anti-reflective coating (scan area 10 μm × 10 μm); (**c**) profiles of the ≈45 μm and ≈95 μm diameter micro-lenses before and after the deposition of the anti-reflective coating.

The surface morphology of the microstructures was measured before and after the deposition of the AR coating. As shown in Figure 11a,b, AR coating reduced the surface roughness from 3.4 nm to 2.6 nm. The observed decrease in surface roughness can be attributed to the ability of the ALD coating to fill in the surface grooves and irregularities, resulting in a smoother substrate surface. Multiple studies [45,46,47] have also reported that ALD coating smoothens the surface.

Further, the focal lengths and profiles of the micro-lenses were evaluated before and after the deposition of AR coating. Optical profilometry measurements showed no difference in the micro-lens height after the deposition (Figure 11c). The focal length measurements were performed using an identical procedure described in Section 3.3. As can be seen from Table 3, AR coating increases the focal length by 1.5–3%. Stress in the AR coating was found to be tensile, with a value of 127 MPa. The first layer of the AR coating was TiO_2_ and a small increase in the focal length of the micro-lens was observed after the deposition due to stress of the AR coating.

The stress of the optical coatings changes the curvature of the flat substrate [43,48]. In this study, a similar tendency was observed, in that the stress of the AR coating affects the geometry of the micro-lenses. This knowledge is important for the design of the final element.

## 4. Conclusions

A quantitative study was performed on the deposition of anti-reflective coating on hybrid organic–inorganic polymer microstructures using the atomic layer deposition technique. First, the optical characteristics of the spin-coated and thermally polymerized SZ2080™ film were evaluated. Then, the influence of titania and alumina thin films on micro-lens geometry were analyzed. The findings showed that the focal length of the micro-lenses decreases by 7.5–8% after Al_2_O_3_ deposition and increases by 2–7% after TiO_2_ deposition. This suggests that for precision applications, primary lens geometry needs to be accommodated to the expected geometry change after applying the coating.

Furthermore, the anti-reflective coating deposited via atomic layer deposition decreased the reflection from 3.3% to 0.1% at a wavelength of 633 nm for one surface of SZ2080™. Fabricated anti-reflective coating improved the transmittance of the micro-lenses by 3.7% without significantly affecting their geometry. The results also indicate that the top-layer surface roughness of the microstructures was slightly reduced after all the depositions. The findings prove the feasibility of ALD coatings on laser 3D lithography-made micro-optics with sub-100 micrometer dimensions employing hybrid organic–inorganic photonic materials.

## Figures and Tables

**Table 1:** Optimized parameters for deposition of Al_2_O_3_ and TiO_2_.

Material	Precursor Pulse Duration, s	Purge Duration, s	O_2_ Plasma Pulse Duration, s	Purge Duration, s	Plasma Power, W
TiO_2_	0.15	15	6	15	200
Al_2_O_3_	0.02	8	6	8	100

**Table 2:** The stress of the single-layer coatings, the focal length of the micro-lenses before deposition (*f*_0_) and after the deposition (*f*) and coating-induced change in the micro-lens focal length (Δ*f*).

Coating	Stress, MPa	Lens Diameter, μm	*f*_0_, μm	*f*, μm	Δ*f*, %
Al_2_O_3_	135	50	187 ± 7	172 ± 2	−8 ± 2
		90	184 ± 6	170 ± 9	−7.5 ± 3
TiO_2_	86	45	142 ± 4	152 ± 5	7 ± 4
		80	156 ± 3	159 ± 5	2 ± 1

**Table 3:** The stress of the anti-reflective coating, the focal length of the micro-lenses before deposition (*f*_0_) and after the deposition (*f*) and coating-induced change in the micro-lens focal length (Δ*f*).

Coating	Stress, MPa	Lens Diameter, μm	*f*_0_, μm	*f*, μm	Δ*f*, %
AR coating	127	45	182 ± 7	185 ± 6	1.5 ± 0.8
		95	190 ± 5	196 ± 5	3 ± 0.5

## Data Availability

The data that support the findings of this study are available within the open access article. Additional data can be provided upon request to corresponding authors.
